# The Implication of NT-proBNP in the Assessment of the Clinical Phenotype of Patients with Type 2 Diabetes Mellitus, Without Established Cardiovascular Disease

**DOI:** 10.3390/biomedicines12122718

**Published:** 2024-11-27

**Authors:** Ioannis Gastouniotis, Christos Fragoulis, Alexis Antonopoulos, Alexandrina Kouroutzoglou, Marina Noutsou, Anastasia Thanopoulou, Christina Chrysohoou, Konstantinos P. Tsioufis

**Affiliations:** 1 First Cardiology Clinic, Medical School, National and Kapodistrian University of Athens, Hippokration General Hospital of Athens, 11527 Athens, Greece; giannisgast@gmail.com (I.G.); antonopoulosal@yahoo.gr (A.A.); alaxandra211764@gmail.com (A.K.); chrysohoou@usa.net (C.C.); ktsioufis@hippocratio.gr (K.P.T.); 2 Diabetes Center, 2nd Department of Internal Medicine, Medical School, National and Kapodistrian University of Athens, Hippokration General Hospital of Athens, 11527 Athens, Greece; mnoutsou@otenet.gr (M.N.); a_thanopoulou@hotmail.com (A.T.)

**Keywords:** diabetes mellitus type 2, heart failure with preserved ejection fraction, natriuretic peptides, cardiopulmonary exercise test, echocardiography, cardiac magnetic resonance imaging

## Abstract

**Background**: Natriuretic peptide (NP) levels have been proposed for characterization and risk stratification of heart failure (HF) among patients with cardiovascular disease (CVD). However, their role in patients with diabetes mellitus type 2 (T2DM) has not been well studied and understood. The aim of this study was to assess phenotypical, functional characteristics and imaging parameters in relation to N-terminal pro b-type natriuretic peptide (NT-proBNP) values in T2DM patients without known CVD that may predispose to overt HF. **Methods:** This was a cross-sectional study of 100 consecutive T2DM patients (mean overall age of 67 ± 9 years, 40% women and 60% men) who were enrolled from the outpatient diabetic clinic. Patients underwent a cardiopulmonary exercise test (CPET), and echocardiographic and cardiac magnetic resonance imaging (CMR); serum NT-proBNP was also measured. **Results:** The mean (standard deviation) NT-proBNP was 149 (±186) pg/mL. Patients in the highest tertile of NT-proBNP values (>107 pg/mL) had lower values of predicted maximum oxygen consumption compared to the lowest quartile (<55 pg/mL) (84% vs. 92%, *p* = 0.018) in the CPET and higher ratio of early diastolic mitral inflow velocity to early diastolic mitral annulus velocity (E/e′) (9.0 vs. 7.2, *p* = 0.05) in echocardiography. Finally, there was a negative correlation between right ventricle end diastolic volume in CMR and predicted maximum oxygen consumption (b = −0.225 ± 0.11, *p* = 0.046). **Conclusions:** NT-proBNP levels seemed to be a useful marker in people with T2DM, as elevated levels reflected ongoing appearance of HF with preserved ejection fraction and were related to CPET and echocardiographic indices of impaired left ventricular diastolic and right ventricular systolic function.

## 1. Introduction

Diabetes mellitus type 2 (T2DM) has been recognized as one of the major non-communicable diseases and is related to high morbidity and mortality of populations, and increasing health costs globally. T2DM increases the risk of progressive atherosclerosis, arterial hypertension, coronary artery disease, and stroke, and has been strongly associated with the occurrence of acute coronary syndrome [1,2].

It has also been recognized that T2DM may precipitate or aggravate heart failure (HF). This occurs due to multifactorial mechanisms—notably, the accumulation of advanced glycation end products, oxidative stress, inflammatory status, reduced intracellular calcium, and changes in microRNAs expression [3,4,5,6]. As T2DM is related to earlier onset of heart failure and other multiorgan complications, the development of risk assessment markers could help with early detection and prevention strategies, promote an early referral system, and promote effective monitoring of those patients [7,8]. Thus, the early identification of subclinical morphological and functional alterations in T2DM patients is important for prioritizing those in high risk of developing HF.

Among several biomarkers, the European Society of Cardiology/European Association for the Study of Diabetes (ESC/EASD) have introduced the use of N-terminal pro b-type natriuretic peptide (NT-proBNP) as a surrogate marker for T2DM patients in the prediction of cardiovascular disease (CVD) risk [9,10,11]. NT-proBNP is a peptide released into the blood primarily by the heart ventricles in response to excessive stretching of heart muscle cells. This typically occurs due to increased pressure within the heart chambers, often associated with heart failure or other cardiac conditions. The exact mechanisms that relate NT-proBNP and T2DM are not entirely understood, but chronic hyperglycemia, insulin resistance, and associated cardiovascular stress are contributing factors. Moreover, there is still a gap in current knowledge regarding the value of NT-proBNP measurement for the prognosis of CVDs among T2DM patients [12,13,14]. Thus, the purpose of this study is to evaluate phenotypical and functional characteristics (echocardiography, cardiopulmonary exercise test, and cardiac magnetic resonance imaging) in relation to NT-proBNP values in T2DM patients without known CVD.

## 2. Material and Methods

### 2.1. Setting

**Figure 1:**
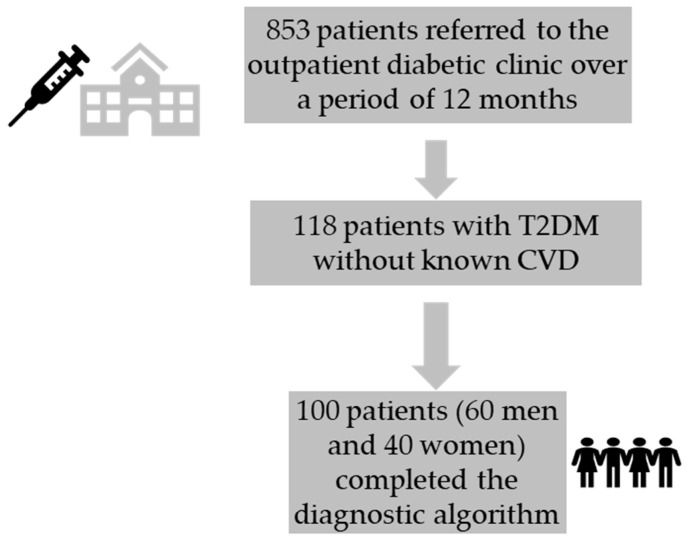
Patient enrollment. CVD: cardiovascular disease, T2DM: diabetes mellitus type 2.

In this cross-sectional study, 100 T2DM patients were enrolled from the outpatient diabetic clinic of our hospital during 2023. Out of 853 patients that visited the diabetic clinic over a time period of 12 months, the screening enrolled 118 patients who met the inclusion criteria. Eighteen patients were excluded for not completing the baseline diagnostic algorithm. The trial design included a 2-year follow-up. The study aimed to discover how functional and imaging assessments could prognose future CVD events and HF progression in a population with T2DM and not known CVD (Figure 1).

### 2.2. Sample

The study’s sample consisted of 100 T2DM patients without known CVD (60% men, mean age of 67 ± 9 years). Eligibility criteria included age of at least 18 years, with a left ventricular ejection fraction (LVEF) of >50% under optimal therapy for T2DM with controlled levels of glycated hemoglobin (HbA1c). Patients were receiving standard medical therapy for other co-comorbidities. Patients with CVD (coronary heart disease, heart failure, stroke, peripheral arterial disease, aortic disease), chronic inflammatory diseases, and cancer were excluded.

### 2.3. Bioethics

The study conformed to the principles of the Declaration of Helsinki and was approved by the Institutional Ethical Committee; any procedures followed the internal hospital protocols. All subjects signed a written informed consent form.

### 2.4. Measurements

All T2DM patients were instructed to abstain from food and smoking for 6 h, and blood samples were taken for NT-proBNP levels measurement using immunoassay (on a Roche Cardiac point-of-care instrument cobas h232 system). Baseline evaluation included socio-demographic characteristics; clinical phenotype; full medical history, including hypertension, dyslipidemia, and years since first diagnosis of diabetes mellitus; as well as dietary and other lifestyle habits (i.e., smoking status and physical activity). Smokers were defined as those who had currently been smoking at least 1 cigarette/day during the past year. Body mass index (BMI) (kg/m^2^) was estimated by dividing measured weight (kg) by standing height squared (m^2^). Arterial blood pressure was obtained in a sitting position, leaving the participant calm for at least 30 min; those with average systolic/diastolic blood pressure levels ≥140/90 mmHg in two different visits or who were under antihypertensive medication were classified as hypertensives. Hyperlipidemia was defined as a history of hypolipidemic treatment and/or total blood cholesterol level ≥180 mg/dL [15].

### 2.5. Cardiac Ultrasound and Ergometric Parameters

Patients underwent a cardiopulmonary exercise test (CPET) and echocardiographic assessment. Echocardiographic assessment was performed using PHILIPS EPIQ7c equipment with a multifrequency transducer (2.5–4 MHz) and tissue doppler imaging (TDI) technology. LVEF was calculated by the Simpson’s method, while right ventricular systolic function was evaluated upon the systolic component of the systolic tissue doppler of the right ventricle (SRV). Left atrial (LA) maximal volume and global longitudinal strain of the left ventricle (GLPS) were also estimated (biplane modified Simpson method). Additionally, GLPS of the left atrial reservoir and the right ventricle was evaluated. Moreover, left diastolic function, left ventricular (LV) hypertrophy, and measurement of LA and LV diameters and volumes were applicable. The measurements were carried out according to the current recommendations, which are also applicable to strain measurements. The ultra-sonographic examinations were carried out by two cardiologists who had at least 5 years of experience in ultra-sonography in a clinical setting [16].

In cardiorespiratory exercise (ergometric treadmill), the maximum oxygen consumption (VO_2_max) anaerobic threshold was defined, while the ratio of minute ventilation/carbon dioxide production (VE/VCO_2_), ratio of oxygen consumption to heart rate (otherwise oxygen pulse: VO_2_/HR), ventilation (VE), and metabolic equivalents per min/day (METS) achieved were evaluated. Additionally, heart rate and blood pressure responses were estimated. Patients underwent Bruce or modified Bruce protocol according to their physical status.

### 2.6. Cardiac Magnetic Resonance Imaging

Cardiac magnetic resonance imaging (CMR) was performed on a 1,5-Tesla scanner (Philips Ingenia 1.5T MRI system) to acquire cardiac structure and function parameters. Cine images were obtained during breath-holding at end-expiration using a balanced steady-state free precession (SSFP) sequence [repetition time (TR) = 2.81 ms; time to echo (TE) = 1.22 ms; slice thickness = 8.0 mm; flip angle (FA) = 40°/50°; acquisition matrix = 166 × 208 pixels; and field of view (FOV) = 340 × 284 mm^2^]. Approximately 10–15 short-axis images from base to apex were obtained, as well as 4-, 2-, and 3-chamber long-axis images. All images were analyzed using commercially available software Circle Cardiovascular Imaging (Cvi 42).

For volumetric analyses, endocardial and epicardial borders were traced semiautomatically at the end-diastolic and end-systolic phases on the short-axis stacks and manually corrected if needed. Biventricular function parameters, including ejection fraction, end-diastolic volume (EDV), end-systolic volume (ESV), and stroke volume (SV), were automatically calculated. LV papillary muscles were included in the left ventricle mass (LVM) but not in the LV volume. Biventricular volumetric measurements and the LVM were indexed for body surface area. For LV contractility analyses, a stack of short-axis cine images combined with 4-, 2-, and 3-chamber long-axis images were loaded into the feature-tracking module. We delineated LV endocardial and epicardial borders at the end-diastolic phase (reference phase) of all cine images. The software automatically traced the contours throughout the cardiac cycle. Global myocardial strain was calculated as the total deformation of the myocardium from its initial length at the end-diastolic phase to its final length at the end-systolic phase and is expressed as a percentage. Positive and negative signs of myocardial strain [peak strain (PS)] indicate shortening and thickening of the myocardium, respectively. Additionally, aortic distensibility was evaluated by measuring from the automated segmentation of aortic cine cardiovascular magnetic resonance using software Circle Cardiovascular Imaging (Cvi 42) [17].

### 2.7. Statistical Analysis

The sample of *n* = 100 participants was adequate, i.e., achieving 83% statistical power, to evaluate 1 standard deviation changes (two-sided hypotheses) on the tested hemodynamic markers by tertile change of the measured parameters at a 5% significance level of two-sided hypotheses. Continuous variables are presented as mean values ± standard deviation. Categorical variables are presented as absolute values and relative frequencies. Age and sex-adjusted analysis of variance (MANOVA) was used to evaluate associations between mean values of normally distributed continuous variables between NT-proBNP tertiles. The normal distribution of the continuous variables was assessed using the Shapiro–Wilk test. The Kruskal–Wallis test was used as an alternative non-parametric approach in the case of non-normally distributed variables. Correlations between continuous variables were evaluated by the calculation of Pearson’s r-coefficient or the Spearman *rho* correlation coefficients. Since NT-proBNP values were heavily skewed, linear regression models with continuous NT-proBNP values as the outcome could not be estimated; therefore, ordinal logistic regression was applied to evaluate the associations of the various measurements, with NT-proBNP tertiles as the outcome. Effect size measures are presented as odds ratios (ORs) and their corresponding 95% confidence intervals (CIs). All reported *p* values are based on two-sided tests. The SPSS statistical software package version 23.0 (Statistical Package for Social Sciences, SPSS Inc., Chicago, IL, USA) was used for all statistical calculations.

## 3. Results

The mean (standard deviation) and median NT-proBNP were 149 (±186) pg/mL and 75 pg/mL, respectively. Regarding clinical characteristics, obesity prevailed in 14% of the patients, hypertension in 64%, and hyperlipidemia in 82%. Moreover, 22% reported regular physical activity, and 20% were active smokers (Table 1).

Descriptive characteristics of the study sample according to NT-proBNP tertiles are presented in Table 2. Patients in the highest tertile of NT-proBNP values (i.e., >107 pg/mL) had lower values of predicted maximal oxygen uptake (VO_2_ pre %) compared to the lowest quartile (<55 pg/mL) (84% vs. 92%, *p* = 0.018) in the CPET and a higher ratio of early diastolic mitral inflow velocity to early diastolic mitral annulus velocity (E/e′) (9.0 vs. 7.2, *p* = 0.05) in echocardiography.

Patients were classified into 2 groups, with NT-proBNP <125 and >125 pg/mL, and further analysis showed different baseline characteristics. Patients with NT-proBNP >125 pg/mL were older in age (71 vs. 61 years old) and showed higher systolic blood pressure (144 +/− 26 vs. 133.4 +/− 17, *p* = 0.01), lower left ventricular EF (54.5 vs. 57%), and higher VE/VCO_2_ (33.4 vs. 29.5, *p* = 0.01) compared with patients with NTproBNP < 125 pg/mL.

Multi-adjusted ordinal logistic regression analysis revealed that NT-proBNP values >107 pg/mL (highest tertile) were associated with lower LVEF (OR 0.88, 95% CI 0.79–0.97) and a higher VE/VCO_2_ slope (1.26, 1.105–2.263), after adjustments for age, sex, obesity, and smoking. Further analysis revealed that increased E/e′ (*p* = 0.02), decreased SRV (*p* = 0.020), and increased VE/VCO_2_ slope (*p* = 0.044) were associated with NT-proBNP values >125 pg/mL (Table 3).

METs achieved in CPET were positively correlated with LVEF (r = 0.997, *p* = 0.02.). VE was inversely correlated with SRV (r = −0.994, *p* = 0.05); VO2 pre % was positively correlated with LVEF (r = 0.997, *p* = 0.05). Linear regression analysis revealed that LVEF (b = 2.546 ± 0.979, *p* = 0.03), LV strain (b = 1.819 ± 0.859, *p* = 0.07), and SRV (b = 3.875 ± 1.687, *p* = 0.02) were associated with higher VO_2_/HR values. Additionally, right ventricular EDV in CMR was positively correlated with VO_2_/HR (*p* = 0.013); CMR right atrial fraction was positively related with VE in CPET (*p* = 0.043). Linear regression analysis revealed that right ventricle EDV in CMR was positively associated with higher values of the uptake efficiency slope (OUES) in CPET (b = 8.736 ± 2.31, *p* = 0.002) and inversely associated with VO_2_ pre % (b = −0.225 ± 0,11, *p* = 0.046), while right atrial volume in CMR was inversely associated with METs in CPET (b = −0.272 ± 0.152, *p* = 0.05).

## 4. Discussion

This study aimed to assess the phenotypical, functional characteristics and imaging parameters in relation to NT-proBNP values in T2DM patients, who may predispose to overt HF. The study revealed that patients in the highest tertile of NT-proBNP values (i.e., >107 pg/mL) had lower values of predicted maximal oxygen uptake and VE/VCO_2_ slope in CPET, higher E/e′ in Doppler echocardiography, and lower LVEF compared to the lowest quartile (<55 pg/mL). Additionally, CMR right atrial fraction was positively related with VE % and inversely associated with METs in CPET (*p* = 0.043), while linear regression analysis revealed that right ventricle EDV in CMR was positively associated with higher values of the OUES in CPET and inversely associated with VO_2_ pre % in CPET.

The pathophysiological pathways that lead to diabetic cardiomyopathy are not well established and understood. However, it seems that many individual factors contribute together, causing alterations in structural and functional parameters of the heart. Hyperglycemia plays a significant role in inducing oxidative stress, which leads to the production of free oxygen radicals, systemic inflammatory response through cytokine release, and activation of the RAAS, causing myocardial hypertrophy, direct damage on the connective tissue, and fibrosis. Furthermore, it causes disturbances in cardiac energy handling, resulting in contractile dysfunction through metabolic toxicity [18,19,20]. In this study, CPET reflected those metabolic disturbances in energy handling; thus, T2DM patients with increased NT-proBNP levels showed lower VO_2_max values, higher VE/VCO_2_, and lower METs achieved.

Natriuretic peptides (NPs) have been proven useful in the initial diagnosis of patients presenting symptoms of HF and in the clinical management of ones with already established HF [21]. Furthermore, NPs have been proven to have a long-term prognostic role in subjects without clinical overt HF but with risk factors for HF development. In a meta-analysis involving 40 studies and 95,617 participants without history of CVD, NT-proBNP strongly predicted first-onset manifestation of HF while providing risk prediction for stroke and coronary artery disease [7]. The predictive ability of NT-proBNP for coronary artery disease and stroke was greater than high-density lipoprotein cholesterol or C-reactive protein, and NT-proBNP could serve as a multipurpose biomarker in new approaches that integrate HF into CVD primary prevention.

In this study, patients with NT-proBNP >125 pg/mL were older in age and showed higher systolic blood pressure, lower left ventricular EF, and higher VE/VCO_2_ (33.4 vs. 29.5, *p* = 0.01) compared with patients with NTproBNP < 125 pg/mL. However, this biomarker criterion has been controversial because it has been shown that some patients with HF with a mildly reduced or preserved ejection fraction may have an NT-proBNP level below this value, especially in obese patients or at a very acute setting of decompensation when diuretic therapy has already been administered [22,23].

A prospective study revealed that patients with a low NT-proBNP were younger, were less often men, and had a higher body mass index than those with a higher NT-proBNP level [24]. Those patients with a low NT-proBNP had less prevalence of atrial fibrillation, myocardial infarction, diabetes, chronic obstructive pulmonary disease, and anemia but better kidney function. Although patients with a lower NT-proBNP level had less marked echocardiographic abnormalities and were less likely to experience cardiovascular death or HF hospitalization, health status according to a quality-of-life questionnaire was similarly impaired in patients with lower and higher NT-proBNP levels [25].

The characterization of diabetes mellitus as a “cardiovascular disease equivalent” was stated in a Finnish study in which T2DM patients without coronary artery disease events showed a similar coronary mortality as non-diabetic patients who had a previous coronary event. Current guidelines no longer consider diabetes as coronary disease risk equivalent [9]. Thus, CVD risk stratification is assessed through certain tools, like Score2-Diabetes, developed by the European Society of Cardiology, and the Atherosclerotic Cardiovascular Disease Risk Estimator, proposed by the American College of Cardiology and American Heart Association [26]. Both scores estimate the 10-year risk of a diabetic patient to experience a cardiovascular event, and aim to recognize patients at higher risks that can possibly benefit from intensified risk factor modifications at a primary prevention level [9,26,27,28,29]. Recently, the PONTIAC I study revealed that treating higher-risk diabetes mellitus patients with a RAS inhibitor plus a beta-blocker decreases the risk of developing severe heart failure by more than half [7]. The Pontiac study proved that treating these patients with a combination of a high dose of RAS inhibitor and a beta-blocker lowers the risk of aggravating heart problems as well as the number of unplanned hospitalizations [7]. On the other hand, in our study, NT-proBNP levels seem to correlate with a phenotypical profile of T2DM patients with no overt clinical CVD who have more impaired functional capacity with lower METs achieved and a higher VE/VCO_2_ slope, slightly lower EF but with higher left ventricular filling pressures, and higher right atrium and right ventricle volumes in CMR.

In accordance with our clinical approach, in a review article, Paul et al. [30] highlighted that regular monitoring for HF in the management of diabetes was a highly advisable approach to identify HF in its early stage. Alongside the clinical approach for HF indicators on every visit, the annual assessment of NPs in patients over 60 (or younger if there are several risk factors for HF) should be a routine aspect of diabetes care. Early identification of individuals at high risk for HF could slow down or possibly prevent the advancement to overt HF. Implementing this approach could likely enhance patient care before they exhibit symptoms and decrease the number of patients diagnosed upon hospitalization due to acute HF, ultimately lowering morbidity and mortality rates. In addition, an observational study by Sojo-Vega et al. [31] pointed out that subclinical atherosclerosis was independently linked to overall mortality and major cardiovascular events in individuals with type 1 diabetes. Thus, employing carotid and femoral ultrasound in screening for subclinical atherosclerosis might facilitate the re-stratification of CVD risk and enhance treatment strategies. Furthermore, another cross-sectional study in 255 T2DM patients without CAD by Xiao et al. [32] emphasized that in adults with T2DM experiencing early HF, the assessment of subclinical atherosclerosis through carotid artery ultrasound and peripheral vascular exams could be effective. Additional research is necessary to enhance diagnostic and treatment methods for individuals in the early stages before HF develops, as well as to validate the cost-effectiveness of these approaches.

### 4.1. Limitations

This prospective study demonstrates some limitations due to design and methodology. Firstly, as an observational study can be limited by unknown or unmeasured confounding factors, as well as by investigator bias due to the consecutive patients’ enrollment. Secondly, the presented results could not be generalized to other outpatient clinic settings or the primary care facilities of other countries since it was planned and performed in a single institution. However, our institution is a first-line hospital covering the medical needs of a large urban population with expertise in DM, hypertensive disorders, and CVD. One of the main limitations is the small number of patients recruited. We acknowledge that assessing a greater number of participants would strengthen our results. Nevertheless, the 12-month duration of the study recruitment, the power analysis performed for the necessary study cohort, and the holistic clinical and laboratory evaluation of each patient and the detailed data collection strengthen the findings. Finally, our trial was restricted by the fact that pregnant women with T2DM or gestational diabetes were not included, only to avoid further confounding factors in a rather special subpopulation.

### 4.2. Novelties—Future Perspectives

Notwithstanding the small numbers, this study included the comprehensive approach of the participants assessing clinical, functional, imaging, and serum data in a rather underexamined population: patients with T2DM with risk factors but without established CVD. The one-year time-interval recording, and the abundance and adequacy of data, were the strongest innovations of this trial. This analysis is part of a three-year follow-up study to enlighten structural and functional changes in this population and illustrate the baseline phenotypes that are prone to appearance of HFpEF.

In future, randomized controlled trials, including a larger number of participants, would clarify the high-risk characteristics of patients with T2DM.

## 5. Conclusions

As T2DM is not any longer considered coronary artery disease equivalent, the identification of patients’ phenotype that is vulnerable in exhibiting HFpEF becomes important. Measurement of NT-proBNP levels in asymptomatic T2DM patients has shown ability in reflecting co-morbidities and ongoing appearance of HFpEF, although even T2DM patients with low NT-proBNP levels could show adverse clinical outcomes. In this work, which was part of a prospective clinical study, we evaluated the role of NT-proBNP levels in the prediction of cardiac functional and structural characteristics related to ongoing appearance of HF. NT-proBNP levels were related with CPET and echocardiographic indices of impaired LV diastolic and right ventricular systolic function. This and other prospective studies may clarify the high-risk patients’ characteristics. Thus, this trial could lead to the proposal of a prognostic tool for future evaluation of these patients.

## Figures and Tables

**Table 1:** Characteristics of the *n* = 100 T2DM patients.

Age, years	67.1 ± 8.5
Male sex, n (%)	61 (61%)
Body mass index, (kg/m^2^)	27.7
History of hypertension, %	65%
History of hyperlipidemia, %	82%
Exercise, %	22%
Duration of diabetes, years	16.1
Current smoking, %	20%
NT-proBNP > 125 pg/mL, %	28%
E/e′	7.95
Global longitudinal strain of left ventricle	−13.5%
Left atrial volume index, (mL/m^2^)	22.7
Left ventricular mass index, (gr/m^2^)	56.9
LVEF, %	52.9%
Left atrial strain rate, %	30%
Four-chamber right ventricular longitudinal strain, %	−12.6
SRV, (cm/s)	37.8
METs at peak exercise in CPET, (kcal/kg/h)	6.9
Maximum heart rate in CPET	152.8
Maximum systolic blood pressure in CPET, (mmHg)	221
VE in CPET, (L/min)	64.9
VO_2_max in CPET, (mL/kg/min)	24.1
VE/VCO_2_ slope in CPET	32
VO_2_/HR in CPET, mL/min/W	14
VO_2_ pre % in CPET	94.2
OUES in CPET	2.0864
CMR right atrial ejection fraction, %	62.4
CMR right ventricle ejection fraction, %	57
CMR right ventricle EDV, (mL)	145.6

CMR: cardiac magnetic resonance imaging, CPET: cardiopulmonary exercise test, E/e′: early diastolic mitral inflow velocity to early diastolic mitral annulus velocity, EDV: end-diastolic volume, LVEF: Left ventricular ejection fraction, n: population, METS: metabolic equivalents per min/day, NT-proBNP: N-terminal pro b-type natriuretic peptide, OUES: uptake efficiency slope, SRV: systolic tissue doppler of right ventricle, T2DM: diabetes mellitus type 2, VE: ventilation, VE/VCO_2_: ratio of minute ventilation/carbon dioxide production, VO_2_ pre %: predicted maximal oxygen uptake, VO_2_max: maximum oxygen consumption, VO_2_/HR: ratio of oxygen consumption to heart rate (otherwise oxygen pulse).

**Table 2:** Comparison of participants’ characteristics among NT-proBNP tertile levels.

	1st Tertile (<55 pg/mL)	2nd Tertile (55–107 pg/mL)	3rd Tertile (>107 pg/mL)	*p*-Value
Age, years	63.5	68.3	70.25	0.03
Male sex, (%)	67%	45%	69%	0.105
Body mass index, kg/m^2^	27.8	28.3	26.9	0.56
Smoking, current (%)	5%	16.1%	31.3%	0.092
Physical activity, (%)	80%	60%	74%	0.1
History of hypertension, (%)	58.3%	70.9%	65%	0.51
History of hyperlipidemia, (%)	83%	77,4%	81.2%	0.4
Years since T2DM diagnosis	16.9	15.6	16.09	0.81
H_2_FPEF Score	1.74	1.62	3.19	<0.01
Cardiac Ultrasound Parameters				
E/A	0.79	0.76	0.74	0.78
E/e′	7.58	7.46	8.7	0.10
Ejection fraction, %	57.4	57.5	55	0.11
Left ventricle EDV, (mL)	149.6	135.8	122.1	0.31
Left atrial volume index, (mL/m^2^)	20.6	25.5	20.8	0.25
Left atrial strain rate, %	24.8	26.4	23.1	0.86
SRV, (cm/s)	13.1	13	17.1	0.41
Four-chamber right ventricular longitudinal strain, %	−12.1	−16	−10.3	0.06
Right ventricle ejection fraction, %	56	60.7	56.7	0.08
Right ventricle EDV, (mL)	130	124.4	128.9	0.91
Right atrial ejection fraction, %	59	62.7	70	0.37
Global longitudinal strain of left ventricle	−13.7	−14.7	−13.2	0.74
Cardiac Ergometric Parameters				
METs at peak exercise in CPET, (kcal/kg/h)	6.89	15.44	11.65	0.14
VO_2_max in CPET, (mL/kg/min)	31.4	40.1	32.2	0.75
VE predicted %	54.4	55.6	51.3	0.58
VE/VCO_2_ slope	31.3	30.5	30	0.58
OUES slope	1880	1936	2314	0.01
Cardiac Magnetic Resonance Imaging				
Myocardial extracellular volume fraction, %	31.8	61.2	28.5	0.82
Right ventricle EDV, (mL)	116.4	135.8	142.6	0.16
Right ventricle ejection fraction, %	57.8	56.5	59.2	0.52
Global longitudinal strain of left ventricle, %	−15.8	−16.6	−15.9	0.76

CPET: cardiopulmonary exercise test, E/A: early-to-late diastolic transmitral flow velocity, E/e′: early diastolic mitral inflow velocity to early diastolic mitral annulus velocity, EDV: end-diastolic volume, METS: metabolic equivalents per min/day, NT-proBNP: N-terminal pro b-type natriuretic peptide, OUES: uptake efficiency slope, SRV: systolic tissue doppler of right ventricle, T2DM: diabetes mellitus type 2, VE: ventilation, VE/VCO_2_: ratio of minute ventilation/carbon dioxide production, VO_2_max: maximum oxygen consumption. Statistical significance *p*-value < 0.05.

**Table 3:** Results from multi-adjusted logistic regression models that evaluated the association of clinical cardiac markers in relation to NT-proBNP levels.

Independent Factor	2nd vs. 1st NT-proBNP Tertile	3rd vs. 1st NT-proBNP Tertile
E/e′ ratio, per 1 unit	0.92 (0.72, 1.19)	0.22 (0.01, 60.94)
VE/VCO_2_ slope, per 1 unit	1.01 (0.87, 1.17)	1.19 (1.04, 1.38) **
Right ventricular ejection fraction, per 1%	1.58 (1.07, 2.33) *	1.10 (0.85, 1.40)
LVEF, per 1%	1.10 (0.90, 1.35)	1.00 (0.83, 1.20)
T1 native blood ms, per 1	1.02 (0.99, 1.03)	1.02 (0.99, 1.03)

E/e′: early diastolic mitral inflow velocity to early diastolic mitral annulus velocity, LVEF: Left ventricular ejection fraction, NT-proBNP: N-terminal pro b-type natriuretic peptide, VE/VCO_2_: ratio of minute ventilation/carbon dioxide production. T1 refers to T1 mapping in cardiac magnetic resonance imaging. Results are presented as ORs and 95% CIs. All models are adjusted for age, sex, BMI, smoking habit, and physical activity level. ** *p*-value < 0.01, * *p*-value < 0.05.

## Data Availability

All data raw and analyzed are available to anyone’s request.

## Abbreviations

ACC/AHA	American College of Cardiology and American Heart Association
ASCVD	Atherosclerotic Cardiovascular Disease
CAD	Coronary artery disease
CI	95% confidence intervals
CMR	Cardiac magnetic resonance imaging
CPET	Cardiopulmonary exercise test
CVD	Cardiovascular disease
E/A	Early-to-late diastolic transmitral flow velocity
E/e′	Early diastolic mitral inflow velocity to early diastolic mitral annulus velocity
EDV	End-diastolic volume
eGFR	Estimated glomerular filtration rate
ESC/EASD	European Society of Cardiology/European Association for the Study of Diabetes
ESV	End-systolic volume
FA	Flip angle
FOV	Field of view
GLPS	Global longitudinal strain of the left ventricle
HbA1c	Glycated hemoglobin
HFpEF	Heart failure with preserved ejection fraction
HF	Heart failure
LA	Left atrial
LV	Left ventricular/ventricle
LVEF	Left ventricular ejection fraction
LVM	Left ventricular mass
METS	Metabolic equivalents per min/day
NP	Natriuretic peptides
non-HDL	Non-high-density lipoprotein
NT-proBNP	N-terminal pro b-type natriuretic peptide
OR	Odds ratio
OUES	Uptake efficiency slope
PS	Peak strain
RAAS	Renin–angiotensin–aldosterone system
RAS	Renin Angiotensin System
SRV	Systolic tissue doppler of right ventricle
SSFP	Steady-state free precession
SV	Stroke volume
TDI	Tissue doppler imaging
T2DM	Diabetes mellitus type 2
TE	Time to echo
TR	Repetition time
VE	Ventilation
VE/VCO2	Ratio of minute ventilation/carbon dioxide production
VO_2_max	Maximum oxygen consumption
VO_2_ pre %	Predicted Maximal Oxygen Uptake
VO_2_/HR	Ratio of oxygen consumption to heart rate (otherwise oxygen pulse)
